# Association of placental chorangiosis with pregnancy complication and prenatal outcome: a case-control study

**DOI:** 10.1186/s12884-021-03576-0

**Published:** 2021-01-30

**Authors:** Homeira Vafaei, Zinat Karimi, Mojgan Akbarzadeh-Jahromi, Fatemeh Asadian

**Affiliations:** 1 Maternal-Fetal Medicine Research Center, Shiraz University of Medical Sciences, Shiraz, Iran; 2 Obstetrics & Gynecology department, School of Medicine, Shiraz University of Medical Sciences, Shiraz, Iran; 3 Pathology Department, School of Medicine, Shiraz University of Medical Sciences, Postal code/ P.O. Box: 34786-71946, Shiraz, Iran; 4 Department of Medical Laboratory Sciences, School of Paramedical Sciences, Shiraz University of Medical Sciences, Shiraz, Iran

**Keywords:** Chorangiosis, Perinatal outcomes, Neonatal outcomes

## Abstract

**Background:**

Chorangiosis is a vascular change involving the terminal chorionic villi in the placenta. It results from longstanding, low-grade hypoxia in the placental tissue, and is associated with such conditions as intrauterine growth restriction (IUGR), diabetes, and gestational hypertension in pregnancy. Chorangiosis rarely occurs in normal pregnancies. However, its prevalence is 5–7% of all placentas from infants admitted to newborn intensive care units. The present study was aimed at determining the association of chorangiosis with pregnancy complications and perinatal outcomes.

**Methods:**

In this case-control study, 308 chorangiosis cases were compared with 308 controls (with other diagnoses in pathology) in terms of maternal, placental, prenatal, and neonatal characteristics derived from the medical records of participants retrospectively. R and SPSS version 22 software tools were used, and the statistical significance level was considered 0.05 for all the tests.

**Results:**

Preeclampsia, diabetes mellitus, maternal hemoglobin, maternal hematocrit, C/S, oligohydramnios, fetal anomaly, dead neonates, NICU admissions were significantly higher in the chorangiosis group OR = 1.6, 3.98, 1.68, 1.92, 2.1, 4.47, 4.22, 2.9, 2.46, respectively (*p*-value< 0.05 for all). Amniotic fluid index, birth weight, cord PH amount, 1st, and 5th Apgar score was lower in the chorangiosis group OR = 0.31, 1, 0.097, 0.83, 0.85, respectively (*p*-value< 0.05 for all). Moreover, fundal placenta, retro placental hemorrhage, perivillous fibrin deposition, calcification, and acute chorioamnionitis were higher in the chorangiosis group OR = 2.1, 11.8, 19.96, 4.05, and 6.38 respectively, (*p-*value< 0.05). There was a high agreement between the two pathologists, and the power of the study was estimated at 99%.

**Conclusion:**

Although chorangiosis is an uncommon condition, it is associated with a higher incidence of perinatal and neonatal morbidity and mortality. Therefore, it should be considered an important clinical sign of adverse pregnancy outcomes and should be reported in the pathology evaluation.

## Background

The placenta, as the largest organ of fetal origin, plays an essential role in fetal development and function from implantation to delivery and affects maternal function during pregnancy. In this regard, histological examination of the placenta provides a wide range of specific information about the nature, cause, and timing of injuries to both the fetus and mother in cases with poor obstetrical outcomes [1–3].

Since oxygen transfer between the mother and fetus is just made through the placenta, its pathological feature indicates current and/or previous chronic fetal hypoxia [2, 4, 5]. Sometimes, villous capillary lesions of the placenta develop as compensatory attempts to improve chronic fetal hypoxia. These lesions form a heterogeneous group of possibly interrelated alterations [6, 7].

Normal chorionic villi should contain fewer than five capillaries in ten high-power microscopic fields, and larger numbers defined as hypervascularity. Extreme villous hypervascularity is known as chorangiosis, characterized by more than 10 capillaries in more than 10 terminal chorionic villi in several areas of the placenta [8].

Chorangiosis is found in 5–7% of all placentas from infants admitted to newborn intensive care units (NICU) [9], and it has to do with increased neonatal morbidity as well as mortality. Furthermore, the prevalence of chorangiosis is higher when some other acute or chronic hypoxia-related placental lesions are present [10–14].

There are limited studies on chorangiosis; its pathogenesis is not precisely exact but believed to result from chronic hypoperfusion and hypoxia [9]. Various maternal, fetal, and placental conditions have been suggested as playing a role in the development of chorangiosis [15], which can be classified into three major categories, namely preuterine, uterine and postuterine patterns of chronic hypoxic placental injury [5, 16]. Preuterine conditions include maternal hypoxemia resulting from low oxygen pressure in the ambient environment (e.g., pregnancies at high altitudes), maternal anemia, high levels of air pollution, maternal smoking, multiple pregnancies, and maternal diabetes mellitus. The uterine pattern of chronic hypoxic placental injury also intervenes in the development of choragiosis. By uterine here, we mean the maternal portions of the uteroplacental circulation, mainly its myometrial and decidual segments, which are the contributing factors to pathologic placental changes. Preeclampsia and late-onset fetal growth retardation are typical examples of the uterine pattern of hypoxia. Finally, primary villous changes, typically referred to as the postuterine (postplacental) pattern of chronic hypoxic placental injury, result in reduced intake of oxygen from the intervillous space. This is often seen in subsets of fetal growth retardation and preeclampsia, and fetal thrombotic vasculopathy (only focally) [5, 16].

Given that chorangiosis is associated with fetal, maternal, and placental disorders, this study is designed to review 308 cases with chorangiosis over a 4-year period to determine the association of chorangiosis with perinatal outcomes.

## Material & Methods

### Study design

This is a case-control study conducted through April 2014 to March 2018. The cases and controls were chosen from the placentas sent to the Pathology Department of Hazrat Zeinab Hospital, affiliated to Shiraz University of Medical Sciences. All placentas had been sent for histopathological examination at the discretion of obstetricians due to high-risk pregnancy, operative delivery, poor condition of the neonate, or gross placental abnormalities.

Selection criteria: Inclusion criteria included the placentas of women aged 15–50 years with gestational age ≥ 20 weeks and singleton pregnancy. Exclusion criteria included incomplete medical records.

### Placenta tissue sampling

All the placentas which arrived at the pathology department were weighted after removal of the cords and membranes. Multiple sections were taken from each placenta, including two sections from the umbilical cord and one from the membrane roll, at least three sections from placental parenchyma (one near umbilical cord insertion and two from central two-third). The tissue was fixed in 10% buffered formalin, embedded in paraffin, and stained with hematoxylin-eosin. Chorangiosis was diagnosed based on the criteria established by Altshuler in 1984 [8], defined as the presence of a minimum of ten villi, each with ten or more vascular channels present in ten or more non-infarcted areas of at least three different placental regions when using an × 10 ocular Lenz. Additionally, other placental pathologies such as calcification, fibrin deposition, retroplacental hemorrhage, and membrane and cord pathology were checked [17]. Reporting of the chorangiosis was done by two pathologists who read the pathology slides and decided on the presence or absence of chorangiosis in the placentas.

### Data collection

Maternal, placental, prenatal, and neonatal features of the participants were retrospectively derived from the medical records of mothers whose placentas had been sent to the pathology department. The variables were maternal features, including maternal age, gestational age, gravid, parity, history of background diseases, hematological indices, and rout of delivery. Fetal features included fetal growth (IUGR, is an estimated fetal weight below the 10th percentile for gestational age) [18], amniotic fluid index, fetal anomaly, and abnormal fetal karyotype. Neonatal features included gender, survival, birth weight (kg), cord PH, first and fifth Apgar scores, and NICU admission. Pathology features included placenta pathology, decidua pathology, membrane pathology, and cord pathology. To control the potential confounding effects of maternal age, gestational age, gravid, and parity, adjusted regression tests were applied in the analysis phase.

### Statistical analyses

Regarding type I error and type II error equal to 0.05 and 0.20, respectively, and based on IUGR association with chorangiosis and the case-control ratio of one, the minimum number of sample size in each group was determined using Cochran’s Sample Size formula. To control the exogenous variables not recorded for the patients as well as the latent confounders, and enhance the power of the study and validity of the results, an attempt was made to take all the cases occurring during April 2014 and March 2018 from a total of 2500 placentas. Finally, at the end of the analysis, a power analysis was performed to estimate the power of the study based on the study results using the followings:

$$ power=\varphi \left\{\frac{\Delta  }{\sqrt{\raisebox{1ex}{${p}_1{q}_1$}\!\left/ \!\raisebox{-1ex}{${n}_1$}\right.+\raisebox{1ex}{${p}_2{q}_2$}\!\left/ \!\raisebox{-1ex}{${n}_2$}\right.}}-{z}_{1-\raisebox{1ex}{$\alpha $}\!\left/ \!\raisebox{-1ex}{$2$}\right.}\frac{\sqrt{\overline{p}\overline{q}\left(\raisebox{1ex}{$1$}\!\left/ \!\raisebox{-1ex}{${n}_1$}\right.+\raisebox{1ex}{$1$}\!\left/ \!\raisebox{-1ex}{${n}_2$}\right.\right)}}{\raisebox{1ex}{${p}_1{q}_1$}\!\left/ \!\raisebox{-1ex}{${n}_1$}\right.+\raisebox{1ex}{${p}_2{q}_2$}\!\left/ \!\raisebox{-1ex}{${n}_2$}\right.}\right\},q=1-p,\kern0.5em \overline{p}=\frac{p_1+k{p}_2}{1+k} $$

p1, p2 = proportion of groups 1 and 2, respectively.

Δ = |p2-p1| = absolute difference between two proportions.

n1 = sample size for group 1.

n2 = sample size for group 2.

α = probability of type I error.

z = critical Z value for a given α.

K = ratio of sample size for group 2 to group 1.

Φ () = function converting a critical Z value to power.

Continuous and discreet variables were described by mean ± standard deviation and frequency and relative frequency, respectively. Kolmogorov-Smirnov test of normality, t-test, X^2^ test, and logistic regression adjusted on maternal age, gestational age, gravid, and parity were used. Kappa statistics were calculated to evaluate how accurate and valid the reporting of the choriongiosis was. Moreover, a power analysis was applied to estimate the power of the study. R and SPSS version 22 software tools were used, and the statistical significance level was considered 0.05 for all the tests.

## Results

There were 308 cases diagnosed with chorangiosis, and 308 other placenta complications controls in the study. The maternal age and gestational age were lower in the chorangiosis group (29.4 ± 5.8, 30.3 ± 5.48 and 35.09 ± 4.10, 36.5 ± 3.49, respectively, and *P*-value< 0.05 for both). However, gravid and parity did not differ significantly between groups (*p*-value> 0.05 for both). The maternal characteristics of the participants are compared in Table. 1.

**Table 1:** Maternal features of 308 chorangiosis cases and the control group from April 2014 to March 2018

Baseline characteristics	Chorangiosis group (***n*** = 308)	Control group (***n*** = 308)	***P***-value
**Maternal age (Mean ± SD)**	29.4 ± 5.82	30.3 ± 5.48	0.04
**Gestational age (Mean ± SD)**	35.09 ± 4.10	36.5 ± 3.49	< 0.001
**Gravidity (Mean ± SD)**	2.26 ± 1.33	2.32 ± 1.29	0.58
**Parity (Mean ± SD)**	1.42 ± 0.82	1.43 ± 0.73	0.89
**Maternal diseases (n, %)**			
**Hypertension**	44 (14.3%)	32(10.4%)	0.14
**Preeclampsia**	58 (18.8%)	39(12.7%)	0.03
**Diabetes mellitus**	50 (16.2%)	15(4.9%)	< 0.001
**Other diseases (n, %)**			
**Depression**	3 (7.1%)	2(10%)	0.92
**Hypothyroid**	24 (57.1%)	13(65%)	
**Minor thalassemia**	6 (14.3%)	2(10%)	
**Multiple sclerosis**	1 (2.4%)	0	
**Asthma**	1 (2.4%)	0	
**Lexi Fredrich syndrome**	1 (2.4%)	0	
**Epilepsy**	1 (2.4%)	0	
**Systemic Lupus Erythematosus**	5(11.9%)	3(15%)	
**Hematological indices (Mean ± SD.**			
**Hemoglobin**	12.33 ± 1.36	11.71 ± 0.88	< 0.001
**Hematocrit**	36.58 ± 3.58	35.04 ± 2.47	< 0.001
**Rout of delivery (n, %)**			
**C/S**	274 (89.0%)	239(77.6%)	< 0.001

Preeclampsia, diabetes mellitus, maternal hemoglobin, maternal hematocrit, and C/S were higher in the chorangiosis group OR = 1.6, 95% C. I (1.03–2.49), OR = 3.98, 95% C. I (2.13–4.45), OR = 1.68, 95% C. I (1.44–1.96), OR = 1.92, 95% C. I (1.13–1.26), and OR = 2.1, 95% C.I(1.33–3.33), respectively. However, hypertension, and other maternal diseases were not significantly different between the two groups (*p*-value> 0.05 for all).

The fetal features of 308 cases from the chorangiosis group and those of the control group are provided in Table. 2.

**Table 2:** Fetal features of 308 chorangiosis cases and the control group from April 2014 to March 2018

Fetal feature	Chorangiosis group (***n*** = 308)	Control group (***n*** = 308)	***P-***value
**IUGR (n, %)**^**a**^	79(25.6%)	27(8.8%)	< 0.001
**Amniotic fluid index (n, %)**			
**Normal amniotic fluid index**	246(79.9%)	288(93.5%)	< 0.001
**Oligohydramnios**	54(17.5%)	12(13.9%)	
**Polyhydramnios**	8(2.6%)	8(2.6%)	
**Fetal anomaly (n, %)**	29 (9.4%)	7(2.3%)	< 0.001
**Abnormal karyotype in fetus (n, %)**	2 (0.6%)	0	0.09

^a^Intra Uterine Growth Restriction (IUGR) is an estimated fetal weight below the 10th percentile for gestational age, based on sonography

The amniotic fluid index was lower in the chorangiosis group OR = 0.31, 95% C. I (0.18–0.54), and oligohydramnios cases were higher in the chorangiosis group OR = 4.47, 95% C. I (2.29–8.72). Moreover, polyhydramnios cases did not differ between the groups (*p*-value> 0.05). Fetal anomaly cases were higher in the chorangiosis group OR = 4.22, 95% C. I (1.49–11.94). However, abnormal karyotype in fetuses did not differ between the groups (*p-*value> 0.05).

The perinatal and neonatal features of 308 babies from the chorangiosis group and those of the babies from the control group are provided in Table. 3.

**Table 3:** Perinatal and neonatal features of 308 babies from the chorangiosis and control groups from April 2014 to March 2018

Perinatal & neonatal feature	Chorangiosis group (***n =*** 308)	Control group (***n =*** 308)	***P-***value
**Gender (n, %)**			
**Male**	179(58.1%)	177(57.5%)	0.87
**Survival (n, %)**			
**Dead**	42 (13.6%)	17(5.5%)	< 0.001
**Birth weight (Kg) (mean ± s.d)**	2372.06 ± 911.4	2780.34 ± 867.365	< 0.001
**Actual birth weight < 10th percentile(n, %)**	38(12.3%)	21(6.8%)	0.02
**PH amount (mean ± s.d)**	7.24 ± .09	7.25 ± 0.06	0.04
**Apgar score at 1 min (mean ± s.d)**	7.09 ± 2.56	8.17 ± 1.84	< 0.001
**Apgar score at 5 min (mean ± s.d)**	8.42 ± 2.55	9.30 ± 1.74	< 0.001
**NICU Admission (n, %)**	140 (45.5%)	68(22.1%)	< 0.001

Gender did not differ between the groups. However, dead neonates and NICU admissions were higher in the chorangiosis group OR = 2.9, 95% C. I (1.58–5.28), and OR = 2.46, 95% C. I (1.62–3.72), respectively. Moreover, birth weight, PH amount, 1st Apgar score, and 5th Apgar score were lower in the chorangiosis group, OR = 1, 95% C. I (0.999–1), OR = 0.097, 95% C. I (0.011–0.86), OR = 0.83, 95% C.I(0.76–0.90), and OR = 0.85, 955 C.I(0.78–0.94), respectively.

Placenta, decidua, membrane, and cord histopathological examination of 308 chorangiosis cases and the control group are presented in Table. 4.

**Table 4:** Placenta, decidua, membrane, and cord histopathological examination and placental site of 308 chorangiosis cases and the control group from April 2014 to March 2018

Placental Features	Chorangiosis group (*n* = 308)	Control group (*n* = 308)	*P*-value
**Placenta site**^**a**^			
Posterior placenta	141(45.8%)	187(60.7%)	0.001
Anterior placenta	117 (38.0%)	99(32.1%)	
Lateral placenta	11(3.6%)	0(0%)	
Fundal placenta	34(11.0%)	18(5.8%)	
Placenta previa	5(1.6%)	4(1.3%)	
**Placenta Pathology**			
Retro Placental Hemorrhage	107(34.7%)	14(4.5%)	< 0.001
Perivillous Fibrin deposition	66(21.4%)	4(1.3%)	
Calcification	32(10.4%)	8(2.6%)	
Accessory placenta	1(0.3%)	0	
Large placenta	9(2.9%)	3(1%)	
**Decidua Pathology**			
Inflamed	4(13.3%)	1(20%)	0.85
Thick wall blood vessel	1(3.3%)	0	
Calcification	25(83.3%)	4(80%)	
**Membrane Pathology**			
Meconium staining	8(17.8%)	7(53.8%)	0.009
Acute chorioamnionitis	37(92.2%)	6(46.2%)	
**Cord Pathology**			
Single umbilical artery	5(38.5%)	2(66.7%)	0.56
Abnormal cord insertion	5 (38.5%)	1(33.3%)	
Acute funisitis	3(23.1%)	0	

^a^According to ultrasonography report

In total, a relationship was seen between the placenta location and chorangiosis status(*p-*value< 0.001). The fundal placenta was higher in the chorangiosis group OR = 2.1, 95% CI (1.15–3.88). Posterior placenta, however, was lower OR = 0.51, 95% C.I(0.37–0.71), and anterior placenta, lateral placenta, placenta previa did not differ significantly(*p-*value> 0.05, for all). Perinatal outcomes are compared between the chorangiosis and control groups by placental site in Table 5.

**Table 5:** The comparison of perinatal outcomes of chorangiosis and control groups by placenta site

Placental site	outcome	Chorangiosis group(n, %)	Control group(n,%)	*p*-value
Posterior placenta	IUGR(*N* = 187)	169(90.4%)	18(9.6%)	< 0.001
Posterior placenta Anterior placenta	C/S(*N* = 141)	122(86.5%)	19(13.5%)	0.009
	Neonatal death(*N =* 187)	173(92.5%)	14(7.5%)	0.04
	NICU(*N =* 141)	75(53.2%)	66(46.8%)	< 0.001
	1stminute Apgar score (mean ± s.d)	7.37 ± 2.18	7.97 ± 2.05	0.012
	IUGR(*N =* 117)	85(72.6%)	32(27.4%)	< 0.001
Anterior placenta Fundal placenta	C/S(*N =* 117)	106(90.6%)	11(9.4%)	0.05
	Neonatal death(*N =* 117)	99(84.6%)	18(15.4%)	0.002
	NICU(*N =* 117)	61(52.1%)	56(47.9%)	< 0.001
	1^st^ minute Apgar score (mean ± s.d)	6.77 ± 2.84	8.44 ± 1.55	< 0.001
	5th minute Apgar score (mean ± s.d)	8.07 ± 2.77	9.50 ± 1.55	< 0.001
	1st minute Apgar score (mean ± s.d)	6.71 ± 3.14	8.71 ± 0.46	0.01
Fundal placenta	5th minute Apgar score (mean ± s.d)	7.9 ± ±3.3	9.77 ± 0.42	0.001

In posterior and anterior placenta cases, IUGR, C/S, neonatal death, and NICU were higher in the chorangiosis group compared to the control group OR = 3.1, 95% C.I(1.67–5.75) and OR = 4.28, 95% C.I(1.87–9.81); OR = 2.16, 95% C.I(1.23–3.87) and OR = 2.14, 95% C.I(1.42–14.99), OR = 2.04, 95% C.I(1.2–4.23) and OR = 5.81, 95% C.I(1.66–20.39), OR = 2.7, 95% C. I (1.69–4.13) and OR = 4.13, 95% C.I(2.2–7.73), respectively. However, they did not differ in fundal placenta, lateral placenta, and placental perivia groups(*p*-value> 0.05 for all). In posterior, anterior, and fundal placenta cases, the first minute Apgar score was lower in the chorangiosis group in comparison to that of the control group OR = 0.87, 95% C.i(0.78–0.97), OR = 0.66, 95% C.I(0.54–0.8), and OR = 0.38, 95% C.I(0.15–0.95). Moreover, just in anterior and fundal placenta cases, the fifth minute Apgar score was lower in the chorangiosis group OR = 0.65, 95% C.i(0.52–0.82), and OR = 0.41, 95% C. I (0.16–0.98) respectively.

Retro placental hemorrhage, perivillous fibrin deposition, and calcification were higher in the chorangiosis group OR = 11.8, 95% CI (6.5–21.45), OR = 19.96, 955 CI (7.12–55.98), and OR = 4.05, 95% CI (1.82–9.01), whereas accessory placenta and large placenta did not differ between the groups. Acute chorioamnionitis was higher in the chorangiosis group OR = 6.38, 95% C. I (2.62–15.49). However, meconium staining did not differ significantly. No significant difference was seen in terms of decidua and cord pathology between the groups. Kappa statistics for chorangiosis reporting by two pathologists was estimated, showing a high agreement of 95%. Moreover, the power of the study was estimated at almost 99%.

## Discussion

The etiology of chorangiosis is not well-known, but it is hypothesized that chronic hypoperfusion or tissue hypoxemia is the leading cause of placenta chorangiosis. According to this theory, hypoxia induces vascular growth factors expression, probably leading to an excessive villous capillary growth and a high proliferative activity of connective tissue [4, 19]. In this study, we reviewed the pathology results of all placentas sent to the Pathology Department over a four-year period to look for evidence of chorangiosis.

In our study, the gestational age was significantly lower in the chorangiosis group. Caldarella et al. [20] showed that chorangiosis generally occurs at term, but no increases have been reported to occur at any gestational age. Nevertheless, some authors have reported chorangiosis is more common after 37 weeks of pregnancy [21]. Another study by GÜN et al. [22] found that chorangiosis had been correlated with increased preterm delivery.

Regarding hematological parameters, our study has also identified that the mean of hemoglobin and hematocrit in women with chorangiosis were significantly more than that of the control group. Although anemia can cause chorangiosis [5, 23], several other conditions contributing to chronic long-standing placental hypoperfusion or low-grade tissue hypoxemia may also lead to chorangiosis. Suzuki et al. [24] revealed that low efficiency of oxygen transfer from maternal to fetal circulation, irrespective of maternal condition, results in chorangiosis as it tends to facilitate vascular remodeling to make up for low oxygen supply. They described a case of diamniotic dichorionic twins, only one of whom manifested growth restriction with placental chorangiosis.

The association of chorangiosis with maternal and placental pathological conditions such as preeclampsia, diabetes mellitus, drug ingestion, urinary tract infections, placental abruption, villitis, and umbilical cord anomalies were also previously noted [20, 22, 25].

In agreement with the previous reports, our results demonstrated that some maternal diseases such as preeclampsia and diabetes mellitus were strongly associated with chorangiosis, while other diseases such as depression, multiple sclerosis, hypothyroidism, minor thalassemia, asthma, epilepsy, and systemic lupus erythematous were not. In this regard, Gun and colleagues reported that out of the ten cases with chorangiosis, two patients had preeclampsia, two had thyroid dysfunction, and one patient had multiple sclerosis [22].

It should be noted that diabetes mellitus is a type of chronic oxidative stress (preuterine pattern). Glycemia affects the capillary of chorionic villi by insulin ephrin-B2 expression, a signaling molecule implicated in sprouting. Preeclampsia also is a state of placental chronic hypoxia with a uterine pattern with an early onset. Serum biomarkers of preeclampsia have been detected as early as 7 weeks of gestational age. Maldevelopment of spiral arteries in the first trimester results in placental malfunction and hence poor pregnancy outcomes [5].

In the present study, the fundal placenta appeared to be higher in the chorangiosis group, and the posterior placenta was found to be lower, while the anterior placenta, lateral placenta, and placenta previa did not vary significantly. In posterior and anterior placenta cases, IUGR, C/S, neonatal death, and NICU were higher in the chorangiosis group, but not different in the fundal placenta, lateral placenta, and placental previa groups. In posterior, anterior, and fundal placenta cases, the first minute Apgar score was lower in the chorangiosis group. No specific study was found to report on the association between chrongeiosis and the placental location. However, some studies were done on the association between placental location and pregnancy outcome.

Placenta location is strongly associated with preeclampsia, causing fetal distress during labor, IUGR, and unfavorable pregnancy outcomes [26, 27]. Zia S. found that anterior placental implantation increases the risk of hypertension, gestational diabetes mellitus, placental abruption, intrauterine growth retardation, and intrauterine fetal death induced by pregnancy. Posterior placenta has a significant association with preterm labor [28]. However, Granfors M. et al. reported that fundal and lateral placental locations, as compared with posterior placental location, are linked to preterm birth, small-for-gestational-age birth, as well as increased manual removal of the placenta in nulliparous women who had vaginal deliveries [29].

Additionally, the rate of cesarean sections in cases with chorangiosis was significantly more than that in the control group. This finding was along with the results obtained from previous studies [25, 30]. It is worth mentioning, however, that the present study was done in a tertiary referral center, and that is why the cesarean sections rate is relatively high in both groups.

Placental lesions are considered as one of the main contributors to neonatal mortality and morbidity. Among the placental lesions, those consistent with maternal vascular malperfusion, such as chorangiosis, have the most significant role to play in adverse fetal outcomes [31]. According to our results, neonatal morbidity and mortality in the chorangiosis population such as low Apgar (min 1 and 5), low birth weight, low PH in arterial blood gas, fetal anomaly, death after birth, and NICU care were higher than those in mothers without chorangiosis. Another important aspect of our findings has to do with the association of chorangiosis with IUGR, oligohydramnios, and anterior placenta. Therefore, chorangiosis is associated with high neonatal morbidity and mortality. This is concordant with the results of all studies on chorangiosis, which reported a higher incidence of neonatal morbidity and mortality in chorangiosis population. Gupta and colleagues showed that neonatal morbidity (IUGR, low birth weight, prematurity, and NICU admissions) were high in 15 cases diagnosed with chorangiosis, and there was a strong positive association of chorangiomatous lesions with pregnancy-induced hypertension/preeclampsia [25]. Moreover, another study performed by Gun and colleagues revealed that preterm birth, low Apgar, and NICU admission were most common in mothers with chorangiosis [22].

Our study has also identified that the incidence of other placental abnormalities (such as fibrin deposition, calcification, and retro placenta hemorrhage), decidua, membrane, and umbilical cord abnormalities in the chorangiosis group was significantly more than that in women without chorangiosis. This is similar to what was reported by Gun et al. [22], who showed that placental calcification, perivillous fibrin, chorioamnionitis, umbilical cord knots, intervillous hemorrhage, and umbilical vein dilatation were commonly observed placental findings in chorangiosis cases. This might be due to the low efficiency of oxygen transfer and maternal vascular malperfusion.

A large sample size study investigating various outcomes and pregnancy characteristics has strengthened the current research, thus providing more accurate statistics, identifying outliers that could skew the data, and lowering the margin of error. It helped control on exogenous variables and latent confounders, thereby enhancing the power of the study. Moreover, a broad clinical and histopathological association with chorangiosis was investigated in the present study. However, a retrospective design left us with no choice but investigate only cases with pregnancy complications without including normal pregnancies, which is a limitation in the study. Choosing a control group with other placental pathological diagnoses was because, in line with the guidelines, only placentas with certain indications are routinely referred to the histopathology lab, and we had to select from other diagnostic pathology complications to examine chorangiosis.

## Conclusion

The findings of our study suggest that chorangiosis should be considered as a hypoxia-associated placental lesion that may occur in association with several maternal diseases such as preeclampsia and diabetes mellitus. In conclusion, although chorangiosis is a rare condition, it is associated with higher incidences of perinatal and neonatal morbidity and mortality. Therefore, it should be considered as a meaningful clinical sign of adverse pregnancy outcomes, and should be mentioned in the pathology evaluation.

## Data Availability

The data of this study are accessible by directly contacting the corresponding author at mojganakbarzadeh@yahoo.com

## Abbreviations

GA: Gestational Age
NICU: Neonatal Intensive Care Unit
AFI: Amniotic Fluid Index
IUGR: Intra-Uterine Growth Retardation
SD: Standard Deviation
